# Postoperative Sigmoid Apoplexy: A Rare Entity in Pediatric Gastroenterology

**DOI:** 10.5005/jp-journals-10018-1114

**Published:** 2014-07-28

**Authors:** Muthukumaran Jagannathan, Gautham Krishnamurthy

**Affiliations:** 1 Department of Pediatric Surgery, Government Stanley Medical College, Chennai, Tamil Nadu, India; 2 Department of General Surgery, Government Stanley Medical College, Chennai, Tamil Nadu, India

**Keywords:** Intramural hematoma, Sigmoid colon, Idiopathic, Intestinal obstruction.

## Abstract

An 11-year-old boy underwent ligation of sac for left congenital hydrocele. In the immediate postoperative period, he developed bleeding per rectum and obstructive features. Intramural hematoma of sigmoid colon was detected in diagnostic laparoscopy and confirmed by laparotomy. Sigmoidectomy with colorectal anastomosis was done. Postoperative period was uneventful.

**How to cite this article:** Jagannathan M, Krishnamurthy G. Postoperative Sigmoid Apoplexy: A Rare Entity in Pediatric Gastroenterology. Euroasian J Hepato-Gastroenterol 2014;4(2):110-112.

## INTRODUCTION

Gastrointestinal apoplexy, also known as intramural hematoma of the alimentary tract, is a rare entity posing diagnostic challenge. Intramural hematoma of the alimentary tract is a rare entity posing diagnostic challenge. It can occur from the esophagus to the rectum.^1^ The disease can result from trauma, bleeding dyscrasias, anticoagulant usage or can be idiopathic.^2^ Intramural hematoma of the large intestine is such a rare entity that only 27 cases were reported for a period of 20 years.^2^ Here, we present a case of 11-year-old boy who underwent liga-tion of sac for left-sided congenital hydrocele and in the postoperative period developed intramural hematoma of the sigmoid colon.

## CASE REPORT

**Fig. 1:**
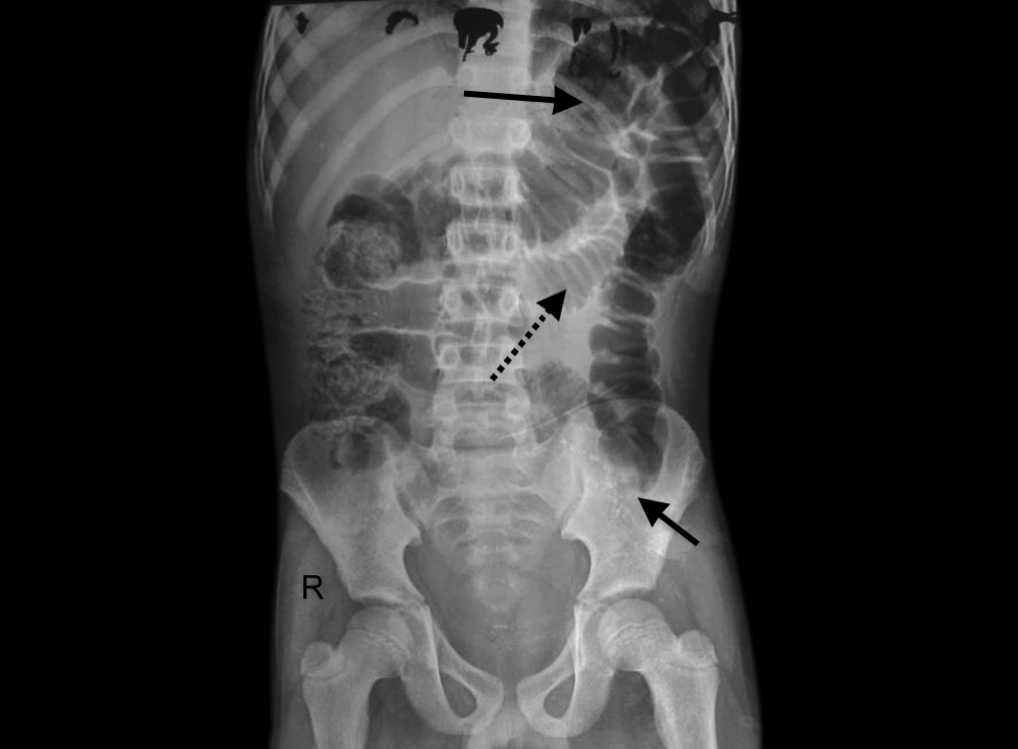
Plain abdomen X-ray taken on postoperative day 1 showing abrupt cut-off at the level of sigmoid colon (small bold arrow) and dilated large intestine (large bold arrow) and jejunum (dotted arrow). There is absence of rectal gas shadow

**Fig. 2:**
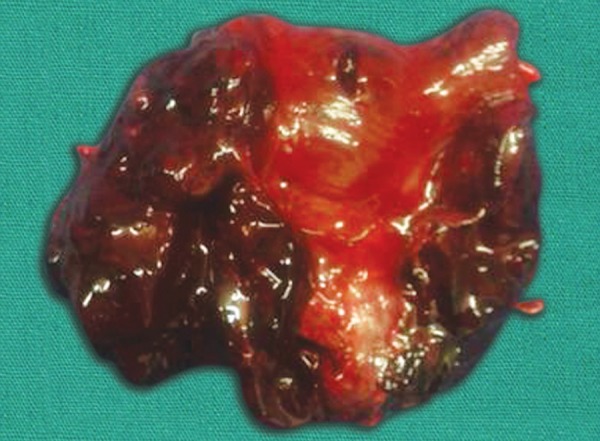
Cut section of the specimen showing a normal mucosa with hematoma confined to submucosal layer of the bowel

An 11-year-old boy was diagnosed with left-sided congenital hydrocele and underwent left processus vaginalis sac ligation under caudal block. His preoperative blood investigation including complete hemogram, bleeding time and clotting time were within normal limits. Patient complained of passing blood admixed with stools in the first postoperative day. On examination, abdomen was soft and per rectal examination revealed bloodstained finger stall. Complete hemogram, platelet count and coagulation profile remained normal. Abdominal X-ray showed dilated small and large intestine with a distal abrupt cut off at the level of sigmoid colon (Fig. 1). Differential diagnosis as per ultrasound abdomen was hematoma or foreign body colon. Patient was started on vitamin K and placed under continuous monitoring. On 2nd postoperative day, patient complained of increased bleeding per rectum (PR) and abdominal distension. With the onset of complete intestinal obstruction, it was decided to proceed with diagnostic laparoscopy instead of planned colonoscopy. Diagnostic laparoscopy revealed dark discoloration of the sigmoid colon measuring 10 cm with grossly dilated descending colon. Laparotomy confirmed the findings. Rest of the bowel was normal. In view of obstructive features, persistent bleeding and limited bowel involvement, sigmoidectomy was done. Intestinal continuity was restored with colorectal anastomosis. Patient was started on orals on 6th postoperative day and passed stools on the same day. Patient recovered completely and was discharged on 12th postoperative day. Coagulation profile at the time of discharge was normal. Histopathological examination showed dissecting hematoma of the sigmoid colon in the submucosal layer (Fig. 2). Computed tomography (CT) angiogram was normal. Patient is on regular follow-up for 8 months and no further episodes of bleeding per rectum.

## DISCUSSION

McLauchlan was the first person to describe the entity of spontaneous intramural hematoma in 1838.^3^ They can be either single or multiple. In majority, the hematoma is found in the submucosal layer splaying the adjacent layers. In contrast to bowel gangrene, in intramural hematoma the viability of the mucosa is preserved. Blunt trauma abdomen is the most common etiological factor. Non-traumatic intramural hematoma associated with anticoagulant usage, coagulation disorders and vasculitis.^4^ Iatrogenic injury causing intramural hematoma of the ascending colon following cholecys-tectomy has been reported.^5^ Primary or ‘idiopathic’ intramural hematoma that occurs without predisposing conditions constitute less than 1% of this disease.^2^

Because of its rarity and benign presentation, the clinical suspicion is seldom made and the diagnosis usually established after radiological imaging or surgical exploration. Varied presentations of this entity include acute conditions like intestinal obstruction, dissection up to serosa resulting in hemoperitoneum and perforation of the weakened bowel wall.

The sonographic appearance of acute intramural hematoma consists of a thickened and echogenic sub-mucosal layer. Contrast-enhanced CT shows intramural hyperdensity of the circumferentially narrowed lumen. Magnetic resonance imaging (MRI) findings include a T2-weighted high-signal intensity ring in the bowel wall surrounding the luminal narrowing.^6^

Idiopathic intramural hematomas of the colon and rectum have rarely been reported.^1^ One study has hypothesized that during straining, the abdominal pressure increases and the rectum also contracts. The contracted rectum increases the intramural pressure, which leads to decreased vascular compliance and rupture, thus causing intramural hematoma of the sigmoid colon and rectum.^1^

Most patients with intramural hematomas present with intestinal obstruction. Conservative therapy is the initial line of management in intramural hematoma because the hematomas will undergo spontaneous resorption and any underlying coagulation disorders engraves the patient for a definitive procedure. The tamponade effect of the hematoma over the bleeder during conservative therapy could curtail bleeding. However, treatment decisions are also influenced by the symptoms and clinical findings. Surgical intervention is required in scenarios where the cause of obstruction is not known or in patients with complete obstruction or who have failed medical management.^7^ The concept of draining the hematoma is not followed as it might increase the risk of bleeding, serious infection and weaken the bowel wall predisposing to perforation.^8^ In our case, in view of the limited non-expanding bowel segment involved and to avoid a second surgery to reverse colostomy, we proceeded with resection of affected bowel and colorectal anastomosis.

The parents were counseled regarding the unrelated postoperative complication and the need for surgical intervention in the setting of acute intestinal obstruction. They have been on regular follow-up as advised.

The etiology of the intramural hematoma could not be clearly identified. Patient did not undergo preoperative bowel preparation in the form of enema. In 1914, Vogel^9^ reported a case of 16-year-old boy with intramural hematoma of sigmoid colon after left inguinal hernioplasty. The author attributed the hematoma to a possible needle prick injury during local anesthesia. In our case, patient underwent sac ligation under intravenous sedation and caudal block. Preoperative and postoperative blood workup indicated no evidence of coagulation defects. The patient did not have clinical features of systemic vasculitis. With histopathological examination showing no evidence of vascular anomalies or vasculitis and angiogram revealing normal study, the possibility of idiopathic intramural hematoma has to be entertained.

## CONCLUSION

Idiopathic intramural hematoma is a rare disorder affecting the gastrointestinal system. Knowledge regarding the predisposing factors and their presence in acute abdomen should arouse clinical suspicion. Imaging modalities play a pivotal role in confirming the diagnosis. Though continuous monitoring is the recommended line of management, surgery is indicated in case of failed conservative approach as in our case.
